# Considerations for the Design and Conduct of a Pharmacovigilance Study Involving Mass Drug Administration in a Resource-Constrained Setting

**DOI:** 10.1371/journal.pntd.0000564

**Published:** 2010-01-26

**Authors:** Demissie Alemayehu, Emma N. Andrews, Paul Glue, Charles A. Knirsch

**Affiliations:** 1 Clinical Affairs, Pfizer, New York, New York, United States of America; 2 Dunedin School of Medicine, Dunedin, New Zealand; World Health Organization, Switzerland

## Introduction

By most estimates, about one billion people worldwide are affected by neglected tropical diseases (NTDs). These diseases are found primarily in developing countries, and those affected are generally marginalized sectors of the population that may not have access to safe water, good hygiene, or adequate medicines. NTDs cause major health problems, and often lead to permanent disability of the victims. Consequently, the social and economic impact of these diseases is massive [1].

With the growing recognition of the deleterious effects of the NTDs, several initiatives are now underway at national and international levels to tackle the problem with the aim of controlling or eliminating them. The drive to contain or eliminate the diseases has further been highlighted in numerous papers [1]–[10], through persistent advocacy of the World Health Organization (WHO) and other institutions [11]–[13], and by funding from government, private, and corporate grants.

An approach that has received wide acceptance in recent years is an integrated strategy that involves mass administration of combination treatments [4], [9]–[15]. This is particularly the case when two or more of the NTDs share a common method of management. The initial emphasis of this approach has been on the seven NTDs: the three soil-transmitted helminthiases (caused by whipworm, hookworm, and roundworm), schistosomiasis, lymphatic filariasis (LF), trachoma, and onchocerciasis. The integrated approach typically involves coordinated use of therapy according to established guidelines, leveraging disease-control activities within the national health system, and active involvement of the community. When the strategy comprises mass drug administration (MDA), the whole endemic population is normally targeted for treatment.

Given the proven efficacy of the individual drugs, an essential facet of the integrated programs is assessment of the safety of the combination therapy in the target population. Accordingly, there is a growing list of studies that have been conducted to evaluate the safety of co-administration of drugs [16]–[19].

Although there is an obvious appreciation of the need to conduct studies to establish the safety of combination drugs in MDA, there has been no public discussion on what guidance may be needed to help researchers in these resource-constrained areas to design, conduct, and analyze such studies. The objective of this policy platform is, therefore, to initiate discussion on this topic, with particular reference to data handling, safety assessment, and other aspects of pharmacovigilance that should be considered to protect the well-being of the target population. Examples will be provided from two studies. The first study [16] was performed in Zanzibar (the “Zanzibar study”) and examined co-administration of ivermectin, albendazole, and praziquantel in children and adults. The second illustrative study pertains to a triple co-administration of azithromycin, ivermectin, and albendazole for the treatment of trachoma and LF (the “trachoma/LF study”). At the time of preparation of this manuscript, preliminary pharmacokinetic (PK) studies for the latter have been reported [15],[17].

While the scope of the this policy platform is the conduct and reporting of MDA studies, it may be worthwhile to note the main features that distinguish such studies from conventional clinical trials for efficacy. In the last section, some relevant aspects of the two types are discussed, with emphasis on compliance requirements and subject inclusion and exclusion criteria.

## Characterizing Potential Pharmacokinetic Interactions

In studying the safety of combination therapy in an MDA setting, it is essential to establish whether interactions among the drugs under study alter the PK profiles of the component agents. Although predictions can be made about the likelihood of metabolic or transporter interactions, such predictions may be difficult when more than two drugs are given, or for older drugs that may lack metabolism or transporter data. A conventional approach to obtaining reliable PK data involves use of carefully designed studies in healthy volunteers. Given the limited availability of centers capable of running PK studies and/or assays in countries with high incidences of NTDs, these may have to be performed elsewhere. When the conduct of such PK studies is impractical, relevant information may be gathered through literature review or simulation and modeling exercises.

For the Zanzibar study [16], PK data in non-infected subjects indicated the absence of pharmacologic interactions among the treatments under consideration, and there was no indication that triple co-administration would enhance their toxicity [19]. Preliminary PK interaction assessment for a trachoma/LF study demonstrates the difficulty of predicting interactions when multiple drugs are co-administered. An initial investigation [15] of the pharmacokinetics of azithromycin both alone and with ivermectin and albendazole demonstrated minimal interaction between azithromycin and albendazole. However, the ivermectin AUC_0→τ_ and C_max_ were increased by 31% and 27%, respectively. A population PK model was developed based on the ivermectin PK data from this study [15] to further characterize the interaction and explore the sources of variability between subjects and across treatments. When the data were simulated 1,000 times for 18,000 subjects exposed, no single value came close to a previously established safety threshold [17].

The results of the PK studies should also be carefully evaluated and used to inform the timing of the follow-up visits for safety assessment.

## Pilot Intervention Phase

Prior to implementing MDA in an endemic area, a pilot study in a limited number of subjects may be carried out to compare the standard of care to the combination therapy. The principal objective of the pilot study would be to establish the safety and feasibility of the combination therapy, and to identify subgroups and events that may require special follow-up during the MDA phase.

The design of the pilot phase may involve cluster randomization, and sample size requirements may need to be justified on statistical grounds. Eligibility may be restricted to consenting residents of endemic sites, excluding subjects with special co-morbidities, children below a specified height, pregnant women, lactating women, and/or women who have given birth within a certain time period (see, e.g., [7] for general guidelines).

For safety follow-ups, both active and passive surveillance approaches will need to be implemented [16]. In particular, as part of the active case detection effort, a process should be in place to manage patients with serious adverse events.

In the Zanzibar study, the pilot trial involved over 5,000 children and adults at two sites. In a trachoma/LF study, the initial trial may consist of an open-label, community-based, randomized, triple co-administration design. The study would enroll eligible children and adults living in an endemic region, with co-administration of azithromycin, ivermectin, and albendazole. The trial duration would be 15 days and may involve two randomly selected villages: village A (standard care as control) and village B (triple therapy).

## Mass Drug Administration Phase

### Subject Eligibility

In a typical MDA study, all consenting residents of selected villages in endemic areas would be eligible for enrollment. However, depending on the formulation and safety profile of the drugs, certain criteria may be used to guide exclusion of subjects from the study [7]. In a trachoma/LF study protocol, for example, subjects may be excluded for one or more of the following criteria: subjects who cannot swallow tablets, subjects who are sick, pregnant women, and lactating women.

### Allocation to Treatment

In general, MDA studies are open-label, and assignment of eligible subjects to treatments should occur sequentially as they are screened for the study at each site. For proper identification (ID), patients may be given ID numbers according to their order of entry into the study.

### Safety Surveillance

Both passive and active measures should be in place to ensure the safety of study participants and the effective assessment of adverse events reported during the study period (see, e.g., [16]).

Passive measures should aim at ensuring rapid identification of, and provision for, medical assistance for treatment emergent signs and symptoms. Health centers with appropriate drug supplies will need to be designated at convenient locations. Planning should also include adequate transportation, for both health care providers and patients, to ensure a rapid response. For serious adverse reactions, a referral system should be established, with referred patients followed up on a daily basis.

Active measures are essential to assess and evaluate the nature and rates of adverse events. Emphasis should be given to serious adverse events, adverse events that may be attributable to the combination therapy, and other adverse events suggested by the pilot phase. However, all treated individuals should be interviewed for occurrences of any side effect using a standardized questionnaire. Efforts should also be made to capture adverse events that may be of interest for special groups, including the elderly, females of childbearing age, children, and patients with co-morbid conditions.

When active case detection is not feasible, which may be the reality in resource-limited areas, every effort must be made to utilize existing health infrastructure to reach patients experiencing adverse events. Coordination with local and regional officials may be essential to ensure rapid communication and response (see, e.g., [20]).

### Subject Withdrawal

Subjects may withdraw from the study at any time at their own request. In a single-dose treatment design, this refers to the situation when a subject does not return for follow-up evaluation. When multiple doses are involved, patients may also be withdrawn at any time at the discretion of the investigator for safety, behavioral, or administrative reasons. If a subject does not return for a scheduled visit, every effort should be made to contact the subject and to document treatment outcome and the reason for withdrawal.

### Adverse Event Reporting

Safety should be assessed for a sufficiently long period of time after drug administration, depending on the PK and safety profile of the individual drugs administered.

An adverse event is defined as any untoward medical occurrence in patients who are administered investigational treatment. The events need not necessarily have a causal relationship with the treatment. Additionally, they may include signs or symptoms resulting from drug overdose, drug withdrawal, drug abuse, drug misuse, drug interactions, drug dependency, extravasations, and exposure *in utero*.

A serious adverse event or serious adverse drug reaction (SAE), on the other hand, is defined as any untoward medical occurrence at any dose that results in death, is life-threatening (immediate risk of death), requires inpatient hospitalization or prolongation of existing hospitalization, results in persistent or significant disability and incapacity, or results in a congenital anomaly or birth defect.

The study protocol should state clearly the reporting procedure for SAEs to local and regulatory authorities, consistent with the local regulations and practice. More specifically, if an SAE occurs, notification should be made within 24 hours of awareness of the event by the investigators. In particular, if the SAE is fatal or life-threatening, notification must be made immediately, irrespective of the extent of available adverse event information.

For all SAEs, the investigator should pursue and provide information that will include a description of the adverse event in sufficient detail to allow for a complete medical assessment of the case and independent determination of possible causality. Information on other possible causes of the event, such as concomitant medications and illnesses, must be provided. In the case of a subject's death, a summary of available autopsy findings must also be submitted as soon as possible. The causality assessment includes the determination of whether there exists a reasonable possibility that the multiple administration caused or contributed to an adverse event, above and beyond that expected by the individual therapies.

## Data Handling and Statistical Considerations

### Data Collection

Simple and convenient data collection tools such as questionnaires, case report forms, or, where possible, electronic data collection devices should be used [21]. The safety data collected should particularly focus on relevant adverse events, including: serious adverse events, adverse events attributable to the combination treatment, and adverse events that may influence compliance with the combination therapy.

As part of the overall planning for the MDA study, it may be worthwhile to establish data centers at convenient locations. Data capture may be facilitated by use of trained personnel or community volunteers. Use of existing health infrastructure is advised to facilitate reporting and assessment of adverse events.

### Sample Size Requirement

In MDA studies, the entire population is normally targeted, and as a result, formal sample size calculations may not be required. However, when there is a need to assess sample size requirements, the unit of randomization (i.e., cluster) should be carefully defined, and the sample size determination and subsequent analysis and data summarization should take into account the clustering. Failure to do so may lead to inappropriate sample sizes and spurious results.

In cluster randomized studies, which may be suitable for the pilot phase, the sample size is a function of estimates of disease prevalence, the cluster size (*m*), and the intra-cluster correlation coefficient (*ICC*) or the design effect (*Deff*). The latter is also known as variance inflation. Loosely speaking, ICC measures the association between pairs drawn from each cluster. For binary data, the kappa coefficient may be used instead of *ICC* [22]. The design effect is the ratio of the number of subjects required using cluster randomization to that required for a design involving simple randomization. It is related to *ICC* as follows:
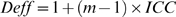



Thus the sample size required for a cluster randomization may be estimated by multiplying by the design effect the number obtained for a simple randomized trial using standard software or a desk calculator.

In addition, the “Rule of Three” may be applied as an aid to assess adequacy of sample size and to enhance understanding of findings of no events as part of the active safety surveillance measures. More specifically, if in *n* subjects no events occur, then an approximation to the upper 95% confidence interval for the true proportion of the event of interest is 3/n. This approximation is reliable for most MDA situations. However, in cluster randomization, the assumption of independence may not be justified (see, e.g., [23]–[26] for relevant references).

### Data Analysis

The analysis strategy for MDA trials should be estimation rather than hypothesis testing. Such studies usually tend to be large, giving statistically significant results even when the results are not clinically significant. When necessary, 95% confidence intervals may be provided along with estimates of treatment effects.

It is essential to pre-specify the main aspects of the analysis strategy either in the body of the protocol or in a separate analysis plan, which must be finalized before data are ready for analysis. The plan should specify at a minimum the primary safety endpoints, criteria for excluding subjects from analysis, and all applicable analytical or data summary methods.

## Administrative Considerations

### Study Monitoring

To ensure adherence to protocol requirements and maximize data quality, the study may have a monitoring plan that can easily be understood and implemented by the study personnel. The plan may describe, among other tasks, roles of personnel responsible for monitoring, the nature and level of monitoring activities, and other data quality assurance steps.

### Safety Monitoring

When there is further concern about exposing a large segment of the population to the combination therapy, the study plan may also include more targeted safety monitoring. This may involve establishing a data and safety monitoring board (DSMB), consisting of independent experts. The DSMB periodically reviews and evaluates the accumulated data, and makes recommendations concerning the continuation, modification, or termination of the trial. In establishing a DSMB for such studies, particular attention should be paid to feasibility and logistical challenges. Operational guidelines for health research sponsors for the establishment and functioning of DSMBs may be found, e.g., in [27].

### Ethical Considerations

The study protocol should be reviewed and approved by an institutional review board (IRB) and ethics committee. For countries where ethics committees are not yet available, it may be necessary to use a non-local committee. In addition, written and oral informed consent should be obtained from study participants. An informed consent by a parent or legal guardian and assent by the child, as appropriate, should also be provided prior to any study-related procedures.

### Training of Study Personnel and Volunteers

Prior to initiation of the study, a meeting should be held involving key study personnel to ensure a thorough and common understanding of the requirements of the protocol. The participants may include health care professionals, opinion leaders, study monitors, and study sponsors. Discussions should focus on eligibility criteria, compliance, safety reporting, and study monitoring. In addition, it is advisable to have presentations on basic concepts of clinical trials and significant aspects of bioethics.

### Special Populations

While the general approach in MDA studies is to enroll all consenting patients with limited exclusion criteria, the safety impact in special populations, including elderly, pediatric patients, and patients with co-morbid conditions, should also be given careful consideration. Since the design of pragmatic studies may not generally be appropriate to assess safety and efficacy in subgroups that require special follow-up and evaluation, other direct and indirect measures may be taken to assess the risk–benefit of treatment. Depending on the subgroup of interest and disease under consideration, reasonable approaches may include epidemiologic studies and meta-analysis (see, e.g., [28],[29]).

Further, in view of the known geographic overlap between the NTDs and such major diseases as malaria, HIV/AIDS, and tuberculosis, treatment strategies eventually may target combination therapies to include these conditions [30]. Therefore, particular attention must be paid to safety assessment in the relevant patient groups.

## Discussion

The wide recognition of the benefits of integrating the control of NTDs has necessitated the need for an effective pharmacovigilance framework to minimize the risk to the population in the affected areas. Given the relatively poor infrastructure in these resource-limited regions, careful planning is essential to gather critical information about the safety profiles of the combination therapy before implementing an MDA program. To ensure a successful outcome, the plan should take into consideration the enormous challenges, in both execution and research, and should be based on an understanding of the local customs and regular health system.

In this policy platform, we highlighted key elements of a MDA study, including PK profiling, trial design, safety data collection and analysis, and other administrative issues. It should, however, be noted that not all aspects of the points considered may be germane for all situations. For example, the establishment of DSMBs or the need for PK profiling may not be feasible or essential depending on the diseases under study, the treatments, or the target population. While the guidance in this paper is primarily intended to raise general awareness of design and logistical issues, any or all of the elements should be implemented with caution and a full evaluation of practicality and relevance.

In addressing issues that are pertinent to the design, conduct, and analysis of MDA studies, it is essential to recognize the features that distinguish such studies from conventional clinical trials for efficacy [31]. While the setting for the latter is typically an ideal condition that permits reliable determination of treatment effect size, pragmatic studies for safety are usually executed under the “usual conditions.” Generally, most consenting adults in endemic areas are included in pragmatic studies, and treatment is administered with more flexibility than in conventional efficacy trials. Unlike efficacy trials in which a placebo may be used as a comparator, in pragmatic studies the control group involves the best available management strategy. Typically, visits may be infrequent and informal in MDA-type studies, with minimal and targeted data collection. Whereas the outcome is generally known to be a direct consequence or surrogate of study drug effect in conventional efficacy trials, in pragmatic studies the goal is to assess safety signals when the drugs are exposed to a wider population in a real-world setting. Unlike in conventional efficacy trials, adherence to protocol by patients or study personnel is not proactively and aggressively monitored in pragmatic studies. It is, therefore, of paramount importance to have in place the active safety surveillance measures discussed earlier to ensure that rare events are not inadvertently missed. Further, requirements for the dissemination of study results for such studies may not be as clearly defined as in the case of clinical trials in the developed world. Therefore, it may be advisable to incorporate in the study protocol the strategy for effective communication of study results.

In most MDA programs, the efficacy of the individual drugs is well established, and studies to evaluate the efficacy of the combination therapy are not often conducted. While PK studies may provide valuable data about drug interactions, they generally are not large enough to conclusively establish the absence or existence of synergy among the drugs involved. Given the added logistical difficulty of collecting reliable data on efficacy and other outcome measures, it may not be feasible to incorporate non-safety data assessment in MDA studies. However, as experience is gained with the conduct of more and more MDA studies in these resource-constrained areas, the plausibility of addressing efficacy issues may eventually need to be tackled.
